# IoT Data Qualification for a Logistic Chain Traceability Smart Contract

**DOI:** 10.3390/s21062239

**Published:** 2021-03-23

**Authors:** Mohamed Ahmed, Chantal Taconet, Mohamed Ould, Sophie Chabridon, Amel Bouzeghoub

**Affiliations:** 1 ALIS International, 4 Rue du Meunier, 95724 Roissy-en-France, France; Mohamed.Ould@alis-intl.com; 2 Samovar, Télécom SudParis, Institut Polytechnique de Paris, 9 rue Charles Fourier, 91011 Evry-Courcouronnes CEDEX, France; Sophie.Chabridon@telecom-sudparis.eu (S.C.); Amel.Bouzeghoub@telecom-sudparis.eu (A.B.)

**Keywords:** IoT, data quality, smart contract, traceability, logistic, sensor, blockchain, supply chain

## Abstract

In the logistic chain domain, the traceability of *shipments* in their entire delivery process from the *shipper* to the *consignee* involves many stakeholders. From the traceability data, contractual decisions may be taken such as incident detection, validation of the delivery or billing. The stakeholders require transparency in the whole process. The combination of the Internet of Things (IoT) and the blockchain paradigms helps in the development of automated and trusted systems. In this context, ensuring the quality of the IoT data is an absolute requirement for the adoption of those technologies. In this article, we propose an approach to assess the data quality (DQ) of IoT data sources using a logistic traceability smart contract developed on top of a blockchain. We select the quality dimensions relevant to our context, namely accuracy, completeness, consistency and currentness, with a proposition of their corresponding measurement methods. We also propose a data quality model specific to the logistic chain domain and a distributed traceability architecture. The evaluation of the proposal shows the capacity of the proposed method to assess the IoT data quality and ensure the user agreement on the data qualification rules. The proposed solution opens new opportunities in the development of automated logistic traceability systems.

## 1. Introduction

In the logistic chain domain, multiple stakeholders need to exchange data about *shipments* transiting from the *shipper* to the *consignee*. The data exchange purpose is to give visibility to all the stakeholders about the *shipments* progress in the logistic chain and trace the path as well as the transport conditions throughout the entire chain.

We refer to the data collected during *shipments* transit as *traceability data*, the system in charge of collecting, saving and sharing those data as *traceability system* and the whole process of data collection and processing as the *traceability process*.

Traditional traceability systems handle traceability data in a central system hosted by one of the stakeholders, which constitutes a risk on the availability of the traceability data (single point of failure). The lack of transparency in the qualification process could also be a source of dispute on the correct application of data handling and qualification rules agreed by all the traceability process stakeholders.

The advent of blockchain technology and smart contracts help develop new traceability systems. Such systems allow stakeholders to achieve the secure and transparent sharing of traceability data, using the blockchain secured and distributed ledger. In addition, smart contracts allow stakeholders to share data handling and decision-making rules, in order to ensure that the same agreed rules are applied by all the stakeholders.

Increasingly, IoT devices are used to automatically collect field data. Those data are used both for traceability purpose and to take automatic decisions, such as the creation of *shipment* incidents, when one or more of the negotiated *shipment* transport conditions are not respected. As a result, the human intervention is limited in the process, as well as process error probability. To automate the traceability process decision making, new traceability system architectures have been proposed in the literature combining smart contracts and IoT (see, e.g., [1,2,3,4]).

However, in the existing smart contracts and IoT traceability systems literature, many of the provided architectures propose to integrate the IoT data directly into the smart contract (see, e.g., [1,2,3,4]). This could lead to unsound decisions taken by the smart contract based on erroneous data collected and sent directly to the smart contract by the IoT data sources. To overcome this issue, we propose to introduce an IoT data qualification process in smart-contracts and IoT-based traceability architectures.

We proposed in [5] to enhance traceability architectures using blockchain, smart contracts and IoT, combined with a lightweight IoT data qualification process. However, in this previous work, the proposed qualification process covered only outlier measure detection, which is only one facet of data quality. Furthermore, we did not compute the qualification at different levels such as the measure and the sensor level. Moreover, the IoT data qualification process was centralized at one stakeholder’s site, and there was no guarantee for the other stakeholders on the correct execution of the agreed IoT data qualification rules. In addition, the stakeholder in charge of the IoT data qualification represented a single point of failure of the architecture on the IoT data qualification part.

To overcome the above limitations, the main contributions of this article are threefold: *(i)* The literature review of IoT data qualification highlights that the data quality of a system is assessed by means of several dimensions. Considering the logistic chain properties, the first contribution is to identify the most relevant IoT data qualification dimensions and provide measurement methods for each of them. *(ii)* To help the stakeholders to get an end-to-end visibility of the data quality and to identify the quality issues causes, the second contribution aims at measuring the data quality at four levels: IoT data events, IoT data sources, shipments and IoT data sources-shipments associations. *(iii)* To ensure the stakeholders agreement on the traceability data, the data qualification rules, and the decisions taken based on the data, such as the creation of incidents, the third contribution consists in integrating the data qualification measurement methods in a traceability smart contract.

The rest of the article is organized as follows. In Section 2, we present the logistic domain context and its requirements through an example use case. Section 3 highlights the main research questions addressed in the paper together with their motivations. Section 4 studies the works related to the IoT data quality and the use of the blockchain to assess this quality. In Section 5, we present the use of the selected IoT data quality dimensions to measure the data quality. Section 6 presents the architecture of the proposed traceability solution. The evaluation of our proposed IoT data quality assessment approach is presented in Section 7. Finally, we conclude in Section 8 and present some future works.

## 2. Medical Equipment Cold Chain Use Case

In this section, we present a business-to-business logistic chain emblematic example. Because of its specific constraints, the medical equipment cold chain is handled by specific transport means. We chose this use case for two reasons: (1) the requirement for transport monitoring; and (2) we worked with an ALIS customer specialized in the production of medical equipment and we were able to discuss with this customer about their traceability needs for this specific cold chain context.

Some of the equipment, such as perishable medical diagnostic kits used in blood tests, needs to be transported under strict conditions with a temperature between a minimum of +2 and a maximum of +8 ${}^{\circ }$C. The non-compliance with this temperature interval may render the medical diagnostic kits unusable. The stakeholders should be notified of any temperature non-compliance.

At least three traceability stakeholders are involved in the traceability system of this medical equipment cold chain: a *shipper* (at the origin of the transport request), a *carrier* (in charge of transport operation) and a *consignee* (the recipient of the transported equipment). In our context, we use the term *shipment* to designate any object entrusted to the *carrier* by the *shipper*, in order to be forwarded to the *consignee*.

The transport condition data are collected through IoT data sources declared by the stakeholders and every IoT data source has its own data communication interval. Among the declared IoT data sources used in our scenario, there is a connected object equipped with sensors that accompanies the *shipments* and that is assigned by the *shipper*.

For visibility and transparency purpose, the stakeholders need to securely share all the traceability data created manually or collected automatically by the IoT data sources. The stakeholders need also to be sure that the traceability data processing conforms to the rules agreed between the stakeholders.

The *shipper* is responsible for the *shipment* creation in the traceability system, with all the data required by the *carrier* for the good execution of the transport operation, such as the origin, destination, transport temperature thresholds and IoT data reception interval. In this scenario, we focus on the management of incidents that could be detected automatically by the traceability system, based on the data sent by the IoT data sources, such as the non-compliance with the negotiated transport temperature interval.

The data received from the IoT data sources will be used to automatically create incidents in the traceability process if necessary. Hence, these data should not be integrated directly into the system. A data qualification process is required to ensure that the IoT data quality is good enough to ensure the proper incidents detection. For this purpose, the stakeholders should have the ability to set the required thresholds for the IoT data quality. Thus, the data that do not meet the quality thresholds requirements could not be used in the traceability process.

The data qualification process has many advantages: it not only provides a quality degree to each *shipment* related IoT event and a performance measure of its associated data source but also helps the users to choose the most trustworthy data source and facilitates the detection of damaged ones in order to repair or replace them.

## 3. Research Questions and Motivations

Based on the above-mentioned use case, we can highlight six main research questions addressed in this article and their motivations: (1) How accurate are the data? In other words, do the data reflect the reality of the *shipment* transport operation? Measuring data accuracy avoids the use of unreliable data. (2) Are the data complete? Indeed, the existence of gaps in the collected data may affect the *shipment* traceability. (3) Are the data consistent? The consistency issue arises when the collected data assigned to a *shipment* comes from several sources with possibly discrepancies leading to incidents. In this case, an agreement could be defined to tolerate a minimum deviation between the data, for example, a gap of 0.5 ${}^{\circ }$C in the temperature may be considered as acceptable. (4) Are the data timely valid? That is, are the data compliant with the receiving window agreed between the stakeholders? The non-respect of this interval may significantly affect the stakeholder’s visibility and the required transparency of ongoing transport operations.

Each above question reflects a facet (dimension) of the quality process that this paper addresses and thus the main contribution of this paper is to propose quality measures for each dimension identified as relevant in our context namely: accuracy, completeness, consistency and currentness. These quality dimensions are defined in the next section.

In addition, to the above quality dimensions questions, there is a concern about quality granularity. (5) How can the system provide different levels of quality: data events, IoT data sources and per *shipment* performances? This high precision quality monitoring facilitates the identification at the right time of the data sources that need to be repaired or removed.

Finally, there is a question concerning transparency. (6) How can the data and the data quality measurement rules be shared securely among the stakeholders to ensure their agreement on the correct application of these rules? To address this issue, we propose to implement the above quality measures into a smart contract, in order to ensure the agreement of all the stakeholders on the correct application of the proposed quality measures.

## 4. Related Works

Data quality is not a recent research topic. The first data quality studies concerned databases. Many data quality aspects have been considered such as the accuracy, consistency and reliability to improve the quality of data inputs into databases and handle databases incompatibility and time critical delivery data [6].

With the advent of the IoT as new data sources, the existing data quality studied aspects needed to be extended to the specificities of those new data sources. The data collected from IoT data sources need to be controlled even more due to the limited capacity of these sources to ensure the security and the quality of their data. The “Never trust user input” should evolve to “Never trust things input”, as stated by Karkouch et al. [7].

Moreover, the emergence of blockchain opens new opportunities for systems that involve multiple stakeholders. The logistic chain domain, which involves multiple stakeholders, provides relevant use cases for this technology [8], especially for traceability purpose [9]. The blockchain promotes the development of smart logistics [10], using smart contracts.

Before providing a literature review, it is important first to define some terms used in the domain of data quality and their meaning in the logistic context.

### 4.1. Data Quality Definitions

Data quality dimensions are attributes representing a single aspect of the data quality, as stated by Richard Y. Wang [11]. In this work, we consider the following data quality dimensions: *accuracy*, *completeness*, *consistency* and *currentness*.

The *accuracy*, as stated by [12], refers to: “the degree to which data has attributes that correctly represent the true value of the intended attribute of a concept or event in a specific context of use”. In our context, it is difficult to know if a received measurement reflects the real *shipment* situation, especially when the *shipment* transport operation is ongoing. However, we can define an accuracy measurement method based on the received measure and the measure source specifications.

The *completeness*, according to [12], corresponds to “the degree to which subject data associated with an entity has values for all expected attributes and related entity instances in a specific context of use”. In our context, the completeness depicts the fact that all the expected events have been received by a data source or a *shipment* according to the update interval agreed by all the stakeholders.

The *consistency*, according to ISO [12], refers to “The degree to which data has attributes that are free from contradiction and are coherent with other data in a specific context of use”. It is also referred to as concordance in some works [13]. In our context, the *consistency* dimension corresponds to the degree of coherence between IoT data events sent by different IoT data sources and related to the same *shipment*.

The *currentness* was defined by ISO [12] as: “The degree to which data has attributes that are of the right age in a specific context of use”. It is also referred to as timeliness, currency, freshness, delay or contemporaneous, in some works [13,14]. In our context, an event is considered of the right age when it is received at the expected time according to the update interval agreed by the stakeholders and defined in the smart contract.

### 4.2. Related Works Study Criteria

The combination of the blockchain smart contracts and the IoT helps in the development of trusted [15] and automated systems. However, the IoT data quality is a hindrance to the development and adoption of this new generation of systems.

In this article, we present the works related to the IoT data quality issue according to three criteria: (C1) the quality dimensions; (C2) the quality levels; and (C3) the use of blockchain smart contracts for data quality management.

#### 4.2.1. Quality Dimensions (C1)

The IoT data quality issue has been addressed using data quality dimensions. For this purpose, the traditional data quality dimensions [11] have been used and adapted to the IoT context needs [13].

The definition of the IoT quality dimensions and their corresponding calculation methods facilitates their usage and application in the target IoT based systems. Due to the lack of works on IoT data using quality dimensions in the logistic chain context, we selected some representative related works from other domains.

Many of the existing works show the interest of using those quality dimensions for IoT data quality handling. In each work, the authors selected the dimensions relevant to their domain and defined the corresponding measurement methods for the selected quality dimensions.

Li et al. [16] defined and measured the currency, availability and validity metrics in a pervasive environment (IoT context) and the problem of data expiration (data no longer usable). It is worth noting that, in the traceability context, the data do not expire. It is important to get all the data for traceability purpose even though the data received late will have a poor currentness quality index.

Sicari et al. [17] proposed a quality-aware and secured architecture handling: accuracy, currentness, completeness and other quality dimensions. A framework for determining the quality of heterogeneous information sources was proposed by Kuemper et al. [18], using the dimensions of accuracy and consistency.

To ensure a real-time data allocation and data quality in multiple partitions collection and storage, Kolomvatsos [19] proposed a real time data pre-processing mechanism, using Fuzzy Logic and handling the accuracy dimension.

In the domain of Ambient Assisted Living (AAL) systems, Kara et al. [20] proposed a quality evaluation model. Their approach is based on the definition and execution of quality metrics and the use of fuzzy logic to evaluate the metrics and decide of the data quality level. In the same precedent domain, Erazo-Garzon et al. [21] defined, measured and evaluated the quality of data collected from an intelligent pillbox, using seven data quality dimensions, among them the accuracy, the completeness, the currentness and the confidentiality.

All the above works use some of or all our required IoT quality dimensions. However, their measurements methods do not meet our needs of dimensions definition and measurement at different levels: data event, data source and *shipment*.

#### 4.2.2. Quality Levels (C2)

In the logistic chain context, the stakeholders need to be provided with a full quality visibility at different levels of the manipulated objects. This is our second criterion (C2). It is helpful for the data quality management and simplifies the investigation in case of discrepancy between the stakeholders IoT data sources. Some works proposed data quality models to handle this issue.

A generic data quality metamodel for data stream management was proposed by Karkouch et al. [22]; in the evaluation of their work, the authors used the accuracy and completeness dimensions. There is also the work of Fagúndez et al. [23] on a data quality model to assess sensors data quality in the health domain, using the dimensions of accuracy, completeness, freshness and consistency.

The above cited models do not meet our context needs. On the one hand, the data sources in our context are reused and affected by different *shipments* in different transport operations. On the other hand, to meet the criterion (C2) in our proposition, we provide the stakeholders with a full visibility of the data quality at different object levels, using an adequate quality model.

#### 4.2.3. Blockchain Smart Contracts for Data Quality Management (C3)

Traceability data need to be shared securely among the stakeholders in order to ensure their agreement on the data quality and the correct application of the agreed data calculation methods. This is our third criterion (C3). The following representative works from the literature propose IoT-blockchain based architectures to handle this issue.

In the domain of crowdsensing platforms, there are many recent works, proposing to use the blockchain in order to improve the quality of the collected IoT data, such as the works of Gu et al. [24], Nguyen and Ali [25], Wei et al. [26], Cheng et al. [27], Huang et al. [28], Zou et al. [29] and Javaid et al. [30]. Their propositions are based essentially on users’ reviews, reputation and reward mechanisms to incentivize the users to improve the quality of their provided data. Those mechanisms are not applicable in the logistic chain context, in which the stakeholders are known and responsible of their provided data.

Casado-Vara [31] proposed an IoT data quality framework based on the use of a blockchain, in the context of smart homes. The proposed solution is limited to the accuracy dimension and does not involve multiple stakeholders, each having its own data sources as is in our context.

In the context of a fish farm, Hang et al. [32] proposed a blockchain based architecture to ensure agriculture data integrity. Their proposed fish farm architecture includes an outlier filter, that removes measurements beyond the expected values. This outlier filter is implemented outside the blockchain, using a Kalman filter algorithm.

Leal et al. [14] proposed a framework for end-to-end traceability and data integrity, in the domain of pharmaceutical manufacturing. They addressed the problem of temporal and multi-source variability using probability distribution methods. In our logistic traceability context, we do not need to estimate sensor measurement data, so we should just report these data as they are sent by sensors. If some data are missed or out of the expected ranges, this results in a quality incident on which the involved stakeholders need to agree.

In our proposition, we implement the data quality measurement methods in a blockchain smart contract in order to ensure a secured sharing and agreement of all the stakeholders on the correct application of the measurement method and the resulting data quality.

### 4.3. Summary of the Related Works Study

To enhance and secure the IoT data quality in the logistic chain, we propose in this article a data quality assessment architecture using accuracy, completeness, consistency and currentness dimensions (C1) in a blockchain smart contract for logistic chain traceability. The proposed architecture provides the logistic chain stakeholders with data quality visibility at different levels (C2) and guarantees the user agreement on the correct quality rules application (C3). Besides, the proposed architecture does not only increase the integrated data quality, but also the stakeholder’s trust and adherence to the resulting automatic decisions.

Table 1 summarizes the selected related works and how they meet the studied three criteria.

## 5. Data Qualification Using Data Quality Dimensions

In this work, the data qualification refers to the definition of data quality measurement methods and the application of those methods on every data received and handled by the smart contract.

We focus on the qualification of traceability IoT data. Because these data are automatically collected and used by the smart contract for the detection of incidents, their qualification is essential for building reliable and automated traceability system.

Thanks to a data quality study adapted to the logistic chain domain, we identified: *(i)* relevant IoT data quality dimensions; and *(ii)* their respective measurement methods.

The IoT data quality model purpose is to be implemented in the traceability smart contract, in order to assess the *shipment* data quality and consequently improve the incidents creation process. Among the quality models proposed in the literature, the model by Karkouch et al. [22] is the closest to our above needs, and we decided to implement and extend this model for the logistic chain domain.

**Figure 1:**
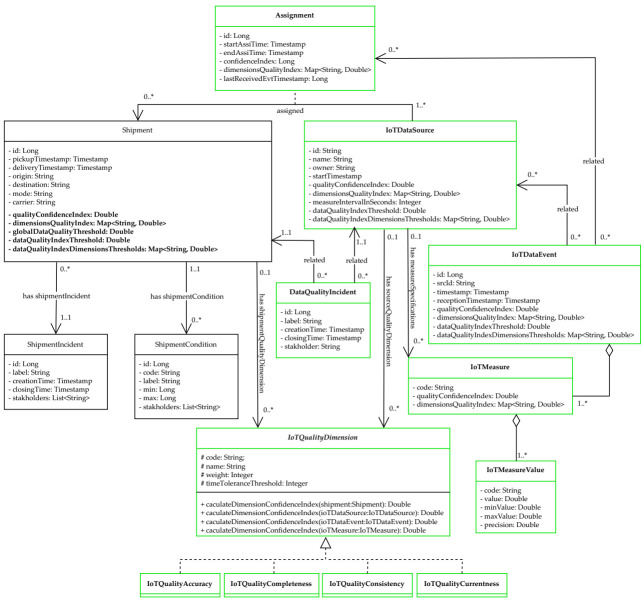
IoT Data Quality Entity class diagram.

As depicted in Figure 1, we added the Shipment entity to collect the data quality at the *shipment* level with its own IoTQualityDimension. Furthermore, for capturing the data quality during the association of the IoTDataSource and the Shipment, we added the Assignment entity which reflects this temporary relationship.

Furthermore, we highlight in Figure 1 all the model entities and attributes added for the quality assessment purpose. The main entity of this model is Shipment which has its own IoTQualityDimension and its own IoTDataSource affected to it through the Assignment entity. It is worth noting that the IoTQualityDimension has a weight attribute defining the importance of the dimension according to the stakeholders needs.

In our context, we need to distinguish different application levels of each dimension, for quality visibility at every object level. The quality index resulting from a dimension application is calculated differently for each dimension related entity in the schema. In some cases (detailed in the next sections), an IoTQualityDimension is not defined for some entities of the schema. For example, the completeness dimension is not defined for IoTDataEvent and IoTMeasure; it is used only for entities with an update time interval constraint such as IoTDataSource and Shipment.

Moreover, we introduce in this model a qualityConfidenceIndex, in order to provide users with an overview of the data quality for the main objects manipulated in our traceability system, which are the IoTDataSource, Shipment, Assignment and IoTDataEvent.

The calculation of the quality index takes into account the weight *W* of dimensions fixed by the users for IoTDataSource and Shipment. We calculate this quality index for the IoTDataSource and Assignment as an average of their *n* IoTDataEvents and *m* IoTQualityDimensions:

(1) $$qualityConfidenceIndex=\frac{\sum_{j=1}^{m}\left(W_{j}*\sum_{i=1}^{n}dimensionQualityIndex_{j_{IoTDataEvent_{i}}}\right)}{\sum_{j=1}^{m}W_{j}}$$

For the Shipment quality index calculation, we use the quality indexes of its related Assignment objects. Regarding the IoTDataEvent, we use the average quality of its related IoTMeasures. The methods used to calculate the IoTQualityDimensions are detailed in the next sections.

The quality thresholds are set by the stakeholders to define the minimum accepted quality index. Values that do not respect this quality will be stored for traceability purpose but will not be used for dynamic incident detection.

To monitor the compliance of the received data according to both the quality threshold and the Shipment transport conditions defined in the smart contract, we added in the model, respectively, the entities: DataQualityIncident and ShipmentIncident. DataQualityIncident results from a non-compliance with the agreed quality thresholds and ShipmentIncident results from a non-compliance with the agreed business transport conditions. For example, consider a IoTDataSource with an interval of possible values from 0 to 50 ${}^{\circ }$C, and monitoring a Shipment with business transport conditions of 2–8 ${}^{\circ }$C. If this IoTDataSource sends a temperature value of 100 ${}^{\circ }$C, this value is considered as non-compliant with the quality thresholds and generates a DataQualityIncident. However, if the sent value is 20 ${}^{\circ }$C, it is considered as non-compliant with the business transport conditions and generates a ShipmentIncident.

In the next subsections, we detail how the dimensions are used to calculate the quality indexes for the different object levels.

### 5.1. Accuracy

The accuracy measurement method is based on the IoTDataSource specifications (sensor measure precision value and sensor minimum and maximum measurable values). Using this method, we can ensure that the received measurement is a possible normal value that can be sent by the concerned IoTDataSource.

Therefore, the received measurement could be used by the traceability smart contract, for example to create an incident, if the received measurement is out of the ranges fixed by the *shipper* for this specific measurement.

In the following subsection, we detail the accuracy calculation method depending on the object level.

#### Accuracy Levels

We identify five accuracy levels: the IoTMeasureValue accuracy $Acc_{MsrVal}$, the IoTMeasure accuracy $Acc_{Msr}$, the IoTDataEvent accuracy $Acc_{Evt}$, the IoTDataSource accuracy $Acc_{Src}$ and the Shipment accuracy $Acc_{Shp}$.

The IoTMeasureValue accuracy as indicated by its name is related to only one value of the IoTMeasure. It is used to indicate if a value of the IoTMeasure is in the range of relevant and acceptable values of this specific IoTMeasureValue, based on the IoTDataSource specifications. For example, consider an IoTMeasureValue *m*, with precision *p*, and $FTh_{min}$ and $FTh_{max}$ are, respectively, the minimum and the maximum possible values given by the IoTDataSource manufacturer.

We calculate the IoTMeasureValue accuracy $Acc_{MsrVal}$ using the following formula:

(2) $$Acc_{MsrVal}=\left\{\begin{array}{cc} 1 & \mathrm{If}\;\left(m-p\right)\geq FTh_{min}\;\mathrm{and}\;\left(m+p\right)\leq FTh_{max}\\ \frac{m-FTh_{min}}{p} & \mathrm{if}\;\left(m-p\right)<FTh_{min}\;\mathrm{and}\;m\geq FTh_{min}\\ \frac{FTh_{max}-m}{p} & \mathrm{if}\;\left(m+p\right)>FTh_{max}\;\mathrm{and}\;m\leq FTh_{max}\\ 0 & otherwise \end{array}\right.$$

The IoTMeasure is composed of *n*IoTMeasureValues, and consequently we calculate the IoTMeasure accuracy $Acc_{Msr}$ as an average of all its IoTMeasureValues accuracies:

(3) $$Acc_{Msr}=\frac{\sum_{i=1}^{n}Acc_{MsrVal_{i}}}{n}$$

The IoTDataEvent accuracy $Acc_{Evt}$ corresponds to an overview of the accuracies of all its related IoTMeasures. This is useful in our context where the IoTDataEvent is considered as a coherent set of IoTMeasures. If this is not the case, the accuracy calculated at the IoTMeasure level can directly be used, and the IoTDataEvent accuracy can be ignored. However, for an IoTDataEvent with n related IoTMeasures, the IoTDataEvent accuracy corresponds to the average of all the IoTDataEvent related IoTMeasures accuracies:

(4) $$Acc_{Evt}=\frac{\sum_{i=1}^{n}Acc_{Msr_{i}}}{n}$$

The IoTDataSource accuracy $Acc_{Src}$ gives an overview of all the IoTDataSource related IoTDataEvents accuracies, it is related to the historic of IoTDataEvents received from the IoTDataSource. In our context, we consider that it is important to take in consideration this historic of IoTDataEvents in the calculation of IoTDataSource accuracy, because it indicates the reliability of the IoTDataSource since it has been deployed and used in our traceability system.

If the users are interested only in the IoTDataSourceIoTMeasures accuracies, the accuracy calculated at the IoTMeasure level could be reused at the IoTDataSource level in order to give them an IoTDataSource accuracy per IoTMeasure. The accuracy of an IoTDataSource corresponds to the accuracy average of all its related IoTDataEvents:

(5) $$Acc_{Src}=\frac{\sum_{i=1}^{n}Acc_{Evt_{i}}}{n}$$

Finally, the Shipment level accuracy emphasizes all the Shipment related IoTDataSource accuracies for the specific time period in which the IoTDataSource is assigned to the Shipment. Every Shipment is considered as an independent transport operation that should have its own accuracy value.

For a Shipment with *n* Assignments to IoTDataSources, the accuracy $Acc_{Shp}$ corresponds to the average of all the Shipment-IoTDataSourceAssignments. For each Assignment accuracy $Acc_{Assign_{i}}$, the number of IoTDataEvents$n_{EvtAssign}$ to be considered in the accuracy calculation, corresponds to the number of IoTDataEvents sent by the IoTDataSource for this specific ShipmentAssignment relationship:

(6) $$Acc_{Shp}=\frac{\sum_{i=1}^{n}Acc_{Assign_{i}}}{n}\;\mathrm{such}\;\mathrm{as}\;Acc_{Assign_{i}}=\frac{\sum_{j=1}^{n_{EvtAssign}}Acc_{Evt_{j}}}{n_{EvtAssign}}$$

### 5.2. Completeness

The completeness measurement method calculates the gap in the data reception for a specific object. It concerns the levels of the IoTDataSource, the Assignment and the Shipment.

#### 5.2.1. Completeness Levels

At the IoTDataSource level, the completeness is calculated based on the source startTimestamp, the source measure interval *I*, the number of received IoTDataEvents *n* from the IoTDataSource and the reception timestamp of the last IoTDataEventlastTimestamp, related to the IoTDataSource:

(7) $$Com_{Src}=\left\{\begin{array}{cc} 1 & \mathrm{If}\;n\geq \frac{lastTimestamp-startTimestamp}{I}\\ \frac{n*I}{lastTimestamp-startTimestamp} & \mathrm{otherwise} \end{array}\right.$$

The Assignment completeness $Com_{Assign}$ means that all the expected IoTDataEvents of the assigned IoTDataSourceSrc, have been received by the Shipment during the IoTDataSource and Shipment association time period enshrined in the smart contract.

Consequently, for the Shipment, the IoTDataEvent frequency is at least one IoTDataEvent per IoT update time interval *I* defined in the smart contract. The $Com_{Assign}$ highlights for the stakeholders the capacity of each IoTDataSource to send all the expected data during its association with a Shipment. This helps the stakeholders to decide on the reusability of the IoTDataSource for further Shipments in the case of a good completeness value or, otherwise, to take over the IoTDataSource in order to identify the completeness source problem.

The $Com_{Assign}$ evolves during the Shipment and the IoTDataSource association time period, and it is recalculated for every new IoTDataEvent reception at the timestamp evtTimestamp, based on the current number of received IoTDataEvents *n*, the Shipment update interval *I*, the IoTDataSource-ShipmentAssignmentstartAssignTime and endAssignTime timestamps.

(8) $$Com_{Assign}=\left\{\begin{array}{cc} 1 & \mathrm{If}\;n\geq \frac{evtTimestamp-startAssignTime}{I}\\ \; & \;\mathrm{and}\;evtTimestamp\in ]startAssignTime,endAssignTime]\\ \; & \;\mathrm{Or}\;\\ \; & n\geq \frac{endAssignTime-startAssignTime}{I}\\ \; & \mathrm{and}\;evtTimestamp>endAssignTime\\ \frac{n*I}{endAssignTime-startAssignTime} & \mathrm{If}\;evtTimestamp>endAssignTime\\ 0 & otherwise \end{array}\right.$$

At the Shipment level, the completeness $Com_{Shp}$ gives an idea of the completeness trend of all the Shipment related IoTDataSources. It is calculated as a $Com_{Assign}$ average of the $n_{Assign}$IoTDataSources assigned to the Shipment:

(9) $$Com_{Shp}=\frac{\sum_{i=1}^{n_{Assign}}Com_{Assign_{i}}}{n_{Assign}}$$

#### 5.2.2. Completeness Incidents

The completeness problem reflects the missing IoTDataEvents. Many reasons could be at the origin of missing IoTDataEvents: network errors, synchronization problems or device malfunctions [33]. If it is not handled, missing data seriously affect the reliability of the data collected through the IoTDataSource.

We propose to generate a completeness incident, if the completeness index of the object fall below the completeness threshold fixed by the stakeholders. The update-missing incident created by the smart contract will also remain there in order to trace the history of data quality problems related to the event IoTDataSource.

### 5.3. Consistency

It is important to calculate the coherence degree between IoTDataEvents and to alert the stakeholders in the case of incoherence detection. The stakeholders should take a corrective action, such as identifying and removing failing IoTDataSource, adapting new threshold values, etc.

The main IoTDataSource in this work is the *shipper* shipment connected object. However, other IoTDataSources could be added by any of the Shipment transport stakeholders. When two or more IoTDataSources assigned to the Shipment monitor the same transport conditions, we calculate the consistency of those IoTDataSources, by comparing their IoTMeasures.

The IoTMeasures comparison takes into account two tolerance thresholds: the time tolerance threshold $Tt_{th}$ and the measure tolerance threshold $Mt_{th}$. Those two thresholds should be defined at the Shipment creation for every IoTDataSource assigned to the Shipment, of course through a mutual agreement between the stakeholders in charge of those IoTDataSources.

#### Consistency Levels

The consistency dimension concerns the levels of IoTDataEvent and Shipment. When an IoTDataEvent$Evt_{i}$ is received from IoTDataSource$Src_{i}$ at a timestamp $Rt_{i}$, and contains a list $Msr_{i}$ of IoTMeasures, we check if there are other IoTDataEvents related to the Shipment and sent by other IoTDataSources, verifying that for each IoTDataEvent$Evt_{j}$, received from IoTDataSource$Src_{j}$ at the timestamp $Rt_{j}$ and containing a list $Msr_{j}$ of IoTMeasures:

(10) $$\left\{\begin{array}{c} Src_{i}\neq Src_{j}\\ |Rt_{i}-Rt_{j}|\;\leq Tt_{th}\\ Msr_{i}\cap Msr_{j}\neq \emptyset \end{array}\right.$$

where IoTMeasures are compared using their codes (see Figure 1).

If there is only one IoTDataSource for the Shipment, or there are no IoTDataEvents verifying the above conditions, then there is no consistency calculation to do. Otherwise, the IoTDataEvent consistency is calculated using the following method:

(11) $$Con_{Evt_{i}}=\left\{\begin{array}{cc} 1\;\forall m\in Msr_{i}\cap Msr_{j},\left|Val_{m_{i}}-Val_{m_{j}}\right|\leq Mt_{th} & Val_{m_{i}}\;\mathrm{is}\;\mathrm{the}\;\mathrm{value}\;\mathrm{of}\;m\;\mathrm{in}\;Msr_{i},\;\mathrm{and}\\ \; & Val_{m_{j}}\;\mathrm{is}\;\mathrm{the}\;\mathrm{value}\;\mathrm{of}\;m\;\mathrm{in}\;Msr_{j}\\ \frac{NbCon_{Evt_{i}}}{NbEvt} & NbCon_{Evt_{i}}\;\mathrm{is}\;\mathrm{the}\;\mathrm{number}\;\mathrm{of}\;\mathrm{events}\\ \; & \mathrm{concordant}\;\mathrm{with}\;Evt_{i},\;\mathrm{and}\;NbEvt\\ \; & \mathrm{is}\;\mathrm{the}\;\mathrm{total}\;\mathrm{number}\;\mathrm{of}\;\mathrm{events}\\ \; & \mathrm{verifying}\;\mathrm{the}\;\mathrm{above}\;\mathrm{consistency}\\ \; & \mathrm{conditions} \end{array}\right.$$

The Shipment consistency $Con_{Shp}$ gives an overview of the Shipment data consistency between all the IoTDataSources related to the Shipment and monitoring the same transport conditions. It is calculated as an average of the Shipment related Assignments consistency: $Con_{Assign}$.

(12) $$Con_{Shp}=\frac{\sum_{i=1}^{n}Con_{Assign_{i}}}{n}\;\mathrm{such}\;\mathrm{as}\;Con_{Assign_{i}}=\frac{\sum_{j=1}^{n_{EvtAssign}}Con_{Evt_{j}}}{n_{EvtAssign}}$$

### 5.4. Currentness

In the logistic traceability context, the currentness dimension may not be critical. Indeed, the most important is to detect incidents, even though the data are received late. However, currentness may reveal incidents concerning data acquisition. Thus, the stakeholders define the Shipment currentness threshold according to the use case.

#### 5.4.1. Currentness Levels

We consider the following currentness levels: IoTDataEvent, IoTDataSource and Shipment.

For an IoTDataEvent$Evt_{i}$, the currentness $Cur_{Evt_{i}}$ is calculated based on the previous IoTDataEvent reception timestamp $t_{i-1}$, the update interval defined in the smart contract *I*, the expected next IoTDataEvent timestamp $t_{i+1}$ which is equal to $t_{i-1}+2*I$ and the current IoTDataEvent reception timestamp $t_{i}$.

(13) $$Cur_{Evt_{i}}=\left\{\begin{array}{cc} 1-\frac{|\left(t_{i-1}+I\right)-t_{i}|}{I} & \mathrm{If}\;t_{i}\in ]t_{i-1},t_{i+1}[\\ 0 & otherwise \end{array}\right.$$

For the Shipments, the interval *I* is a *shipper* requirement that should be met through the sending of an IoTDataEvent to the smart contract, every time that this interval has elapsed. Consequently, the currentness indicates not only the quality of the data but also the meet degree of one of the more important *shipper* requirements defined in the smart contract, the Shipment update interval *I*.

Furthermore, the $Cur_{Evt_{i}}$ at the IoTDataSource level is calculated using the same above method, but it is worth noting that the IoTDataSource has its own update interval that could be different from the Shipment update interval.

Regarding the IoTDataSource, the currentness corresponds to the degree to which the IoTDataSource has met the update interval time requirement, in the entire history of its related IoTDataEvents, including the last received IoTDataEvent.

The currentness dimension helps the users in the choice of the IoTDataSources to be assigned to the Shipment, users will always choose the IoTDataSource with the highest currentness among the available IoTDataSources. The IoTDataSource currentness $Cur_{Src}$ is calculated as the average of all the IoTDataSource related IoTDataEvents:

(14) $$Cur_{Src}=\frac{\sum_{i=1}^{n}Cur_{Evt_{i}}}{n}$$

From the Shipment perspective, the currentness indicates the degree to which the *shipper* update time interval requirement has been met for the Shipment by all its related IoTDataSources, during the Shipment-IoTDataSource association time period. To measure the currentness performance of the Shipment-IoTDataSource association, the currentness calculated for this association $Cur_{Assign}$ is saved in the Assignment object.

The $Cur_{Assign}$ is useful when the Shipment stakeholders need to investigate a low Shipment currentness, as it helps to identify the Shipment related IoTDataSource(s) responsible(s) of the low currentness value. The Shipment currentness $Cur_{Shp}$ corresponds to the average $Cur_{Assign_{i}}$ of all its *n* related Assignment objects. The $Cur_{Assign}$ is calculated as a $Cur_{Evt_{j}}$ average of the $n_{EvtAssign}$IoTDataEvents received from the IoTDataSource for the Shipment, during their Assignment association:

(15) $$Cur_{Shp}=\frac{\sum_{i=1}^{n}Cur_{Assign_{i}}}{n}\;\mathrm{such}\;\mathrm{as}\;Cur_{Assign_{i}}=\frac{\sum_{j=1}^{n_{EvtAssign}}Cur_{Evt_{j}}}{n_{EvtAssign}}$$

#### 5.4.2. Currentness Incidents

There are two currentness control points, the reception of the IoTDataEvent by the stakeholder IS (shipper IS, *carrier* IS or *consignee* IS) and the reception of the IoTDataEvent by the smart contract. In case of non-reception of the IoTDataEvent by the stakeholder IS, this leads to a missing update on the smart contract side.

The IoTDataSource is configured to send an IoTDataEvent every *n* seconds. If this interval has elapsed and no new IoTDataEvent has been received from the IoTDataSource, the situation is considered as a missing update problem.

The missing update is not critical if the IoT update interval $I_{sc}$ of the smart contract is larger than *n* seconds, because the smart contract generally does not wait for a new IoTDataEvent as long as this update interval does not expire.

In contrast, if the update interval is equal to *n* seconds, the stakeholder IS notifies the smart contract in the case of missing data. Once notified, the smart contract assigns a missing update related incident to the IoTDataSource owner. The origins of this kind of incidents are multiple; for example, the IoTDataSource is not able to connect to the IoT network, the IoTDataSource has internal problem or an IoT cloud data platform problem.

## 6. The Distributed Architecture of the Traceability System

This section presents the architecture of the proposed traceability solution and its main components: the blockchain smart contract and the IoT data sources.

**Figure 2:**
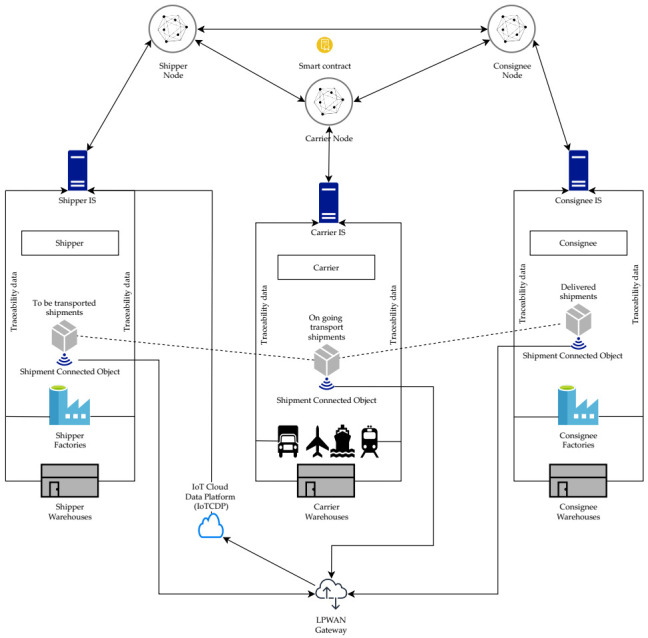
Distributed architecture of the traceability system.

To respond to the identified criteria for secured traceability data, data qualification and transparency, we propose a distributed, secured and trusted architecture, based on the use of blockchain smart contracts, as depicted in Figure 2. The main components of this architecture are a smart contract shared by all the stakeholders and IoT data sources. The arrows in this figure indicate data transmission directions.

For the smart contract component, we chose to work on a Hyperledger Fabric blockchain [34]. It is a permissioned blockchain that presents many advantages in comparison to the other blockchains, among them: a node architecture based on the notion of organization to establish a trust model more adapted to the enterprise context, the support of the Go, Javascript and Java languages for writing smart contracts and a parameterized consensus protocol [5].

The smart contract is installed on the top of a blockchain involving all the stakeholders. This smart contract holds all the rules about the collected traceability data management, the incident management and the IoT data qualification. Those rules have been validated by all the traceability system stakeholders.

IoT data sources are assigned to each *shipment*. They are responsible of the field data collection about the *shipment* transport conditions.

It is worth noting that the three stakeholders depicted in Figure 2 are given as examples. As many stakeholders as needed may be added to this architecture. The addition of stakeholders is enabled by the underlying Hyperledger Fabric- based architecture [34]. In addition, the maximum number of stakeholders in the context of logistic chain is limited. For example, in our use case, this number is of the order of tens stakeholders.

The stakeholders to be added to this architecture are those who need to participate in the traceability process. They are added before the creation of any *shipment* transport operation in which they will be involved.

### 6.1. The Smart Contract

Smart contracts are “trusted distributed applications” [34]. They are secured by the underlying blockchain and the peers consensus mechanism. In the transport traceability context, we need a distributed and secured application to share traceability data among all the traceability process stakeholders and ensure their agreement on the shared data quality and the incidents created based on this data.

We proposed in [5] to implement a lightweight IoT data qualification application and a traceability smart contract handling all the *shipment* transport operation process. The implemented smart contract allowed the stakeholders to define all the transport conditions terms, update the transport status and transport related milestones status, integrating IoT data about the *shipment* transport operation progress and creating both manual or automatic transport related incidents.

The contractual constraints, negotiated between stakeholders, are enshrined in the smart contract, and should be respected by all the stakeholders. Any gap between those constraints and the data provided by a stakeholder results in a non-compliance incident created automatically by the smart contract. The contractual constraints are communicated to the smart contract by the *shipper* system at the *shipment* creation time.

In this article, we extend the traceability proposal presented in [5] to overcome two important limitations. *(i)* The IoT data qualification is centralized at the *shipper* side, and there is a lack of guarantees for the other stakeholders on the good execution of the agreed IoT data quality calculation rules. *(ii)* The lightweight IoT data qualification module is limited to data outlier’s detection.

Therefore, we propose in this work to enhance the IoT data application through the implementation of the quality model presented in Section 5, into the traceability smart contract. This allows ensuring the stakeholders agreement on the correct application of the data qualification rules. The data qualification module is also improved by the integration of the accuracy, completeness, consistency and currentness dimensions.

IoTDataEvents that do not conform to the defined IoT quality model constraints generate DataQualityIncident visible by all the stakeholders. They are not discarded but saved in the blockchain for audit purpose.

As examples of decisions taken automatically by the smart contract based on the received events, Table 2 shows some temperature events values received by the smart contract, their Quality Indexes (QI) and their corresponding decisions. For these examples, we consider multiple IoT data sources with manufacturer temperature specifications interval of [0 ${}^{\circ }$C, 50 ${}^{\circ }$C]. Those data sources are assigned to a *shipment* with a temperature conditions transport interval of [2 ${}^{\circ }$C, 8 ${}^{\circ }$C]. The quality dimensions’ weights are set to 4 for accuracy, 4 for consistency and 1 for Currentness. If the event QI is below the quality index threshold (0.7) a DataQualityIncident is generated for the event. Consequently, the event QI is calculated as follows:

(16) $$EventQI=\frac{4*(AccuracyQI)+4*(ConsistencyQI)+1*(CurrentnessQI)}{9}$$

where 9 is the sum of dimensions’ weights (4+4+1).

### 6.2. The IoT Data Sources

In the proposed traceability architecture, the IoT data could be received from many IoT data sources. Each stakeholder could decide to assign an IoT data source that it owns to a *shipment* in which it has a stakeholder role, at any time during the *shipment* progress in the logistic chain. The only condition to do so is that the IoT data source and the *shipment* have already been created in the smart contract.

The assignment of an IoT data source to a *shipment* is for a limited period. Every data source assigned to a *shipment* sends IoT data about the *shipment* transport conditions at a fixed time interval defined in the *shipment* smart contract instance.

If a data-related incident is detected by the smart contract, it is automatically affected to the IoT data source owner declared in the smart contract. The smart contract has a detailed description of the IoT data source specifications collected at the data source creation in the smart contract. This is a requirement for the correct application of the data quality measures.

The *shipper* in our context has a principal IoT data source which is the shipment connected object accompanying the *shipment*. The role of this object is to collect data about the *shipment* transport conditions, throughout the transport operation.

To send the collected data to the *shipper* IS (Information System), the connected object uses an LPWAN (Low Power Aera Network) network Gateway, which transmits the received messages to the IoT Cloud Data Platform (IoTCDP) before their reception in the *shipper* IS.

The *shipper* IS sends the received messages to the *shipper* node including the connected object id of the messages. This connected object id is used by the smart contract to link the received IoT messages to the right *shipment* in the smart contract. In this context, the data are pushed by the IoT object. The pull/push of data from/to the connected object is out of the scope of our work. The shipment connected object collects data about the *shipment* pickup, transport and delivery conditions.

Each stakeholder could declare other IoT data sources, such as IoT data sources related to factories, warehouses, transport vehicles, etc. In general, every data source that can collect and send automatically measurements about the *shipments* could be declared by the stakeholder as an IoT data source. Moreover, all IoT data sources, except the shipment connected object, help to collect data about the *shipment* conditions in a specific segment of the transport operation. Only the shipment connected object that accompanies the *shipment* continues to collect data about the *shipment* transport conditions during the whole transport operation.

## 7. Evaluation

The objectives of this section are: *(i)* to evaluate the proposed quality measures; *(ii)* to evaluate the impact of the IoT data quality module on the number of created incidents; and *(iii)* to evaluate the impact of the IoT data quality module on the IoT data event insertion time in the blockchain.

We evaluated our proposed quality measures to measure their pertinence and performance. We also monitored the number of quality incidents created to highlight the impact of the quality module. The number of *shipment* incidents was also monitored to emphasize the impact of the quality module on the business decisions.

The IoT data event insertion time in the blockchain was also measured in our tests, firstly with the quality module activated and then with the quality module inactivated, in order to evaluate the impact of our proposed quality module on the data event insertion time and ensure the final users that this time is acceptable while ensuring the quality.

### 7.1. Smart Contract Architecture

For the implementation purpose, we used the same architecture used in our previous work on the traceability using smart contracts and IoT [5]. It is an architecture based on the use of Hyperledger Fabric as the blockchain implementation, with three peers (stakeholders): a *shipper*, a *carrier* and a *consignee*. On the top of this blockchain, we implemented our traceability and IoT data qualification smart contract.

The smart contract used in this evaluation was developed on the top of a Hyperledger Fabric blockchain, using the Fabric Java Framework. We used in this evaluation a Virtual Machine (VM) with the characteristics depicted in Table 3.

Furthermore, we set the Hyperledger Fabric block creation timeout to 1 s and the maximum number of transactions per block to 15. This means that, after the reception of a new transaction, the system will trigger the block creation either after a time wait of 1 s or after a total number of 15 new transactions is reached. In addition, we used in this evaluation the Raft consensus algorithm, with a unique ordering service node [5].

In the existing traceability smart contract [5], we added many new methods such as createDataSource and assignDataSource. The createDataSource method inserts the data source given as input in the blockchain. The assignDataSouce method assigns an existing IoT data source to an existing *shipment*, using their IDs. Based on the quality measures proposed in this article, we updated the addIoTEvent method with the following new functionalities: *(i)* calculate the event quality measures; and *(ii)* update the IoT data source and the quality measures of the *shipments* related to this IoT data source.

### 7.2. Evaluation Experimental Choices

Due to a lack of real data to evaluate the proposed architecture in our use case, we chose to simulate our use case data with a well-known dataset in the IoT domain. The Intel Berkeley dataset is a collection of sensor data, collected by Intel research team in the Intel Berkeley Research lab, between 28 February and 5 April 2004 [35]. An example of the dataset content is depicted in Table 4.

To adapt this dataset to our context, we considered every sensor as an IoT data source. This gives us 54 data sources to be handled. For the *shipments*, we used every 24 h of sensor data collection as a *shipment*, which results in 2052 *shipments* (54 sensors multiplied by 38, the number of data collection days), for the whole dataset.

Furthermore, we considered only the temperature measures in this evaluation because it is the main measure for our use case, but the module could be used to handle any other measure type.

We began the evaluation phase by defining the user’s quality thresholds requirements for all the data sources and *shipments*. We used the same threshold for the data sources, the *shipments* and the four quality dimensions. We made a series of tests by varying the defined threshold, going from 0 (no quality constraints) to 1 (strict quality), to show the impact of those thresholds on the number of created quality and *shipments* incidents.

In Table 5, we establish a classification of data quality indexes for our dimensions and objects. This classification helps in the presentation and the analysis of the evaluation results.

We chose the following weights for the quality dimensions based on their importance for the use case in the context of the medical equipment cold chain: a weight of 4 for the accuracy, the completeness, and the consistency, which are the most important for our users, and a weight of 1 for the currentness, which is not as critical as the other dimensions, as explained in Section 5.

For the *shipment* incidents, we chose an accepted temperature interval of 20 to 25 ${}^{\circ }$C based on the work of Hui et al. [36]. Beyond this temperature interval, if the received event quality is compliant with the *shipment* quality threshold, this event results in a *shipment* incident created for all the *shipments* that have an active assignment relationship with the event data source.

There was no information in the dataset about the sensor’s precision value. Consequently, we chose to set this value to 0.5 ${}^{\circ }$C, which is a recurrent value in the temperature sensors.

In the following evaluation results, we did not take into account the sensor 5 from which we did not see any event. We also ignored some other events with the sensor IDs 55, 56 and 58, because in the dataset reference the number of sensors was only 54, and events coming from the same sensor with the same event number (113,474 events in the dataset).

There were also 355 events in the dataset that we could not parse correctly due to their data presentation errors and 526 incomplete lines, from which we could not get all the event required data. This results in a total of 2,199,327 events integrated correctly in our quality tests, from a total of 2,313,682 events present in the dataset.

We used the event timestamp in the dataset as an event reception timestamp in this evaluation. Moreover, we used this timestamp to order and identify the events, for *shipment* incident creation and closing purpose. The results of this choice were 10,299 duplicated events, because they had the same timestamp as previously received events from the same sensor.

Furthermore, we use the quality threshold to define the stakeholder’s requirement for the quality indexes of events to be integrated in the data source or sent to *shipments*. All the events with a quality index below the defined quality threshold value results in a quality incident and are not used to create *shipment* incidents in case of non-compliance with the agreed transport conditions. If the quality incident is detected by the data source, it will not send the event to its related *shipments*.

### 7.3. Results Concerning the Accuracy, Completeness and Currentness Dimensions

Firstly, regarding the accuracy, the sensors used to collect the Intel Berkeley dataset, a valid temperature value should be in the range of 0–50 ${}^{\circ }$C according to [37], otherwise we consider this temperature as inaccurate.

Regarding the completeness, we used the following parameters: the update interval of 31 s, the maximum timestamp among the already integrated events timestamps, the start IoT data source and the *shipment* start timestamp. We set the IoT data source start timestamp at 28 February 2004 at 00:00:00 am, and, for the *shipment*, the start timestamp is the *shipment* date and the start time set at 00:00:00 am and the end at 11:59:59 pm.

Concerning the currentness, we used the measure interval of 31 s given for the dataset. We used this same update interval for the data sources and the *shipments*. In our tests, we did not consider the difference that could exist between the event reception timestamp and the event production timestamp. This difference could affect the test and need to be addressed in future works.

Table 6 shows the classification of quality results obtained for the sensors (data sources), regarding the different quality dimensions defined in this work and using multiple quality threshold values. Those results show that in the 53 retained sensors: 42 have a good accuracy, 29 have a poor completeness and 29 have a lower currentness.

Regarding the global sensor quality index, most sensors (38) have a low-quality index. If the quality threshold is set to a good quality value (e.g., 0.7), only 15 sensors are usable, and, in the case of threshold of high quality (e.g., 0.9), there is no usable sensor in this dataset.

Thanks to the quality module, all the events with a quality incident problem are not integrated into the *shipments* assigned to the event data source, and this keeps the *shipment* events quality at the level fixed and agreed by all the stakeholders. For example, in the case of Sensor 45, when we set the quality threshold at 1, 9% of the events received from this sensor have not been integrated into the source related *shipments*, due to their quality problems.

In Table 7, we can clearly see the impact of the threshold choice on the percentage of quality incidents. This percentage represents the events that do not respect the agreed quality thresholds. The events are filtered at the data source level according to the selected quality threshold value.

Consequently, the percentage of quality incidents drops from around 25% of the total received events for a threshold at 0.5 to around 21% when the quality threshold was greater or equal to 0.7. The percentage of *shipment* incidents evolution is not linear due to the *shipments* number evolution depending on the selected quality threshold, as depicted in Table 8.

Regarding the *shipments* quality results, it is important to note that there were 421 *shipments* for which we did not receive any event, no matter what the quality threshold value was. This number increases to 821 *shipments*, when we set the quality threshold at 0.5, 0.7, 0.9 or 1, as depicted in Table 8. Consequently, we did not consider those *shipments* in the following *shipment* quality results, because all our quality dimension calculations are based on the events values and timestamps.

Table 9 shows that the percentage of *shipments* with a high accuracy level increase as the *shipments* quality thresholds increases, and this is the same for the currentness. The percentage of events with a poor completeness index increases due to events blocked by the quality threshold at the data source level.

The *shipment* quality index also is improved by the quality threshold increase; for example, we went from 27% of poor data quality *shipments* when the quality threshold was at 0 to only 2%, when the quality threshold was up to 0.5.

### 7.4. Results Concerning the Consistency Dimension

**Figure 3:**
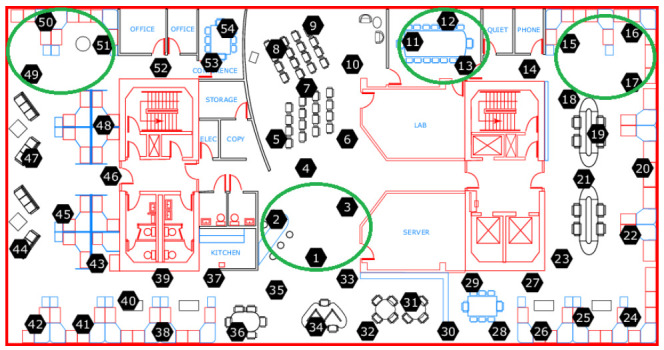
Intel Berkeley sensors arrangement diagram.

For the consistency evaluation, we selected four groups of sensors placed in proximity zones, as depicted in Figure 3: {1, 2, 3}, {11, 12, 13}, {15, 16, 17} and {49, 50, 51}. For each group, we linked each sensor to all its related sensors *shipments* in the same sensors group.

The total number of *shipments* related to the selected groups was 456 (12 sensors multiplied by 38 data collection days). There were 84 *shipments* related to those groups, for which we did not receive any event from the sensors, whatever the quality threshold value. This number increases to 171 *shipments* when we set the quality threshold at 0.5, 0.7, 0.9 or 1, due to the events quality filtering at the data source level.

Furthermore, we set in this evaluation the tolerance time interval to 31 s and the consistency tolerance temperature to 0.5 ${}^{\circ }$C. This means that two events are considered as eligible to the consistency test only when their timestamps difference is lower than 31 a, and they are considered as concordant if their reported temperatures difference is lower than 0.5 ${}^{\circ }$C.

Table 10 summarizes the consistency evaluation results for the selected sensors groups. The group {1, 2, 3} has at least 76% of its *shipments* with a high consistency index. Those results show that the events reported by the group {1, 2, 3} were more concordant than those reported by the other groups.

The consistency results for the selected groups were generally good to high, except for 3% of *shipments* related to the group {1, 2, 3}, when the quality threshold was at 0. This shows the impact of the quality threshold on the consistency quality results.

### 7.5. Impact of the IoT Data Quality Module on the IoT Data Event Insertion

For the smart contract IoT data quality evaluation, and due to our blockchain architecture response time (around 1 s per operation), we selected a sample of 3000 events from the dataset. This sample corresponds to the first 1000 events received from the Sensors 1–3 on 28 February 2004.

The average response time of the addIoTEvent using the 3000 events data sample was around 1.7 s, with an average standard deviation of 0.174 s. When we disabled the quality module, with the same data sample, the average response time of this method drops to around 1.6 s, with an average standard deviation of 0.158 s.

This result shows that our quality module adds only around 0.1 s to the event integration time. The additional quality module cost is acceptable regarding the data quality improvement brought by this module.

### 7.6. Related Works Discussion

As shown in Section 4, the works of Casado-Vara et al. [31], Hang et al. [32] and Leal et al. [14] are the closest to our work.

Casado-Vara et al. [31] proposed a vote method to address the accuracy and the consistency problems. Their vote method is based on the game theory to find a cooperative temperature among all the used temperature sensors. It is not applicable in our context, because we have different data sources owned by different stakeholders, and we need to report all the data sent by those data sources for audit purpose.

In the case of discrepancy between the stakeholder’s data sources related to the same *shipment*, we need to trace this discrepancy, and, if it goes below the fixed quality threshold, a corresponding quality incident is created by the smart contract. However, the vote method in [31] could be used in the very specific case of many *shipments* with similar data sources, the same *shipper*, the same *carrier* and from which we want to have a global measure trend.

Hang et al. [32] proposed a Hyperledger Fabric based architecture. This blockchain implementation choice is perfectly adapted to our B2B use case, and we used the same in our proposed architecture. However, they only addressed the accuracy problem (outlier filtering) using the Kalman filter.

Besides, the standard version of Kalman filter did not meet our needs, because the outlier interval limits are not fixed and evolve according to the received data. This could be problematic when the Kalman filter goes in fail mode, as stated by Berman [38]. The usage of an assisted version of the Kalman filter needs to be explored in future work.

Leal et al. [14] proposed using an Ethereum traceability-based architecture. Their Ethereum choice is justified by the solution monetization goal. However, in our use case, we chose to work with Hyperledger Fabric which does not need any cryptocurrency management and has an organization architecture more adapted to our B2B logistic chain context, in terms of data access levels management.

In addition, Leal et al. [14] addressed the accuracy, consistency and currentness problems using probability distribution methods, but they did not provide further details about their application and evaluation of those methods.

Furthermore, the authors of [14] proposed to filter the data inside and outside the blockchain, which is a good idea, and we already have in our architecture the inside blockchain data filtering. Besides, we need to explore the adding of a data filtering first level outside the blockchain, in future works.

The outside blockchain filtering needs to be done carefully, because it should not prevent the blockchain from getting the required traceability data; although, in some cases these data will be outliers, they need to be traced for further audit purposes.

### 7.7. Conclusions on the Evaluation

This evaluation section demonstrates the pertinence of the proposed IoT data quality module and the impact of this module on the data to be used in the traceability smart contract. The entire data qualification process is executed in a secured and distributed application on which users agree on every datum to be included, on its qualification process and decisions to be taken based on this datum.

It is worth noting that the choice of the quality thresholds has a huge impact on the data filtering process set at the data source level. The events with a quality index below the defined quality threshold will never be sent to the *shipment*. This leads directly to data loss at the *shipment* level. For this reason, stakeholders may prefer selecting a good quality threshold ([0.7,0.9]), rather than a high one ([0.9,1]).

Although the proposed architecture evaluation shows encouraging results, this architecture still needs to be tested in a real-life scenario with more data and stakeholders to get more information about its real performances.

## 8. Conclusions and Future Works

In this article, we propose a distributed architecture and a smart contract to enhance the IoT data quality in the context of logistic traceability. The proposed architecture uses a model of IoT data quality with four main data quality dimensions: accuracy, currentness, completeness and consistency.

We also propose an approach for the calculation of the selected data quality dimensions. The dimensions calculation results are used in our traceability smart contract to set and control the data quality of events, data sources, *shipments* and *shipments* data sources associations.

The proposed architecture ensures the stakeholders agreement on the data quality calculation and application rules, and consequently their trust in the decisions taken automatically by the traceability smart contract. We evaluated our proposed IoT data quality assessment architecture based on an online available dataset, and the results show the relevancy of this architecture.

This work could be extended by evaluating the scalability of the proposition when adding more stakeholders. The approach used to calculate the quality dimensions could be combined with algorithms, such as DBSCAN [39] or an assisted version of the Kalman filter [40], to improve the quality index calculation.

The blockchain data charge could be alleviated by adding in this architecture a first level of data filtering on each stakeholder side. The IoT data sources’ security and interoperability also need to be addressed. Finally, the architecture evaluation needs to be done in a real-life scenario to ensure its performance in the context of logistic chain traceability.

## Figures and Tables

**Table 1:** Related works comparison summary.

	C1 (Quality Dimension)	C2 (Quality Levels)	C3 (Use of Blockchain Smart Contracts for Data Quality Management)
IoT			
Li et al. [16]	Currentness and others	Data	N/A
Sicari et al. [17]	accuracy, currentness, completeness and others	Data and stream window	N/A
Kuemper et al. [18]	Accuracy and consistency	Data and data source	N/A
Kolomvatsos et al. [19]	Accuracy	Data	N/A
Kara et al. [20]	Accuracy, completeness and others	Data	N/A
Erazo-Garzom et al. [21]	Accuracy, completeness, consistency (lack of measurement method), currentness and others.	Data and data source	N/A
IoT Data Quality models			
Karkouch et al. [22]	Accuracy and completeness (in the evaluation)	Data and stream window	N/A
Fagúndez et al. [23]	Accuracy, completeness, freshness and consistency	Data and stream window	N/A
Blockchain and IoT Crowdsensing platforms			
Gu et al. [24], Nguyen and Ali [25], Wei et al. [26], Cheng et al. [27], Huang et al. [28], Zou et al. [29] and Javaid et al. [30]	N/A	N/A	Data quality ensured through reviews, reputations and rewards mechanisms implemented in blockchain smart contracts
Blockchain, IoT and data qualification			
Casado-Vara et al. [31]	Accuracy and consistency	N/A	Accuracy qualified outside the blockchain smart contract and consistency inside it
Hang et al. [32]	Accuracy (outliers filtering)	N/A	Outlier’s filtering outside the blockchain smart contract
Leal et al. [14]	Accuracy, consistency (multi-source variability) and currentness (contemporaneous)	N/A	Data qualification outside and inside the blockchain smart contract
Our proposition	Accuracy, completeness, consistency and currentness	Data, data source, *shipment* and *shipment* data source relationship (equivalent to Stream window)	Data qualified using quality dimensions implemented in a blockchain smart contract

**Table 2:** Smart contract decisions examples.

Received Temperature Events Values	Accuracy QI	Consistency QI	Currentness QI	Event QI	Smart Contract Decision
10 ${}^{\circ }$C	1	1	1	1	Create a ShipmentIncident
−20 ${}^{\circ }$C	0	1	1	0.55	Create an accuracy DataQualityIncident
Many times −20 ${}^{\circ }$C from the same source	0	1	1	0.55	Create an accuracy DataQualityIncident, and finally a completeness DataQualityIncident at the Source-Shipment Assignment level
Event1 of −20 ${}^{\circ }$C from a source 1 and Event2 of 10 ${}^{\circ }$C from a source 2	0 for Event1 and 1 for Event2	0.5 for both	1 for both	0.33 for Event1 and 0.77 for Event2	Create a consistency and accuracy DataQualityIncident from Event1 and a ShipmentIncident from Event2
10 ${}^{\circ }$C received late from one source	1	1	0	0.88	Create a ShipmentIncident

**Table 3:** Test VM characteristics.

Characteristic	Details
OS	Ubuntu 18.04.4 desktop amd64
CPU	4 CPU Intel(R) Core™ i7-8565U
RAM	8 G
Virtual Disk	50 G

**Table 4:** Samples of the Intel Berkeley dataset.

Date	Time	Event ID	Sensor ID	Temperature	Humidity	Light	Voltage
12 March 2004	16:29:04.084098	39302	1	21.8308	43.5855	165.6	2.53812
14 March 2004	15:45:11.669786	44974	2	26.9464	41.814	264.96	2.54901
19 March 2004	19:01:21.094445	59766	3	21.9092	45.1103	39.56	2.44412
…	…	…	…	…	…	…	…

**Table 5:** Quality indexes and thresholds classification.

Data Quality Index and Threshold Interval	Label	Code
[0,0.5)	Poor quality	P
[0.5,0.7)	Low quality	L
[0.7,0.9)	Good quality	G
[0.9,1]	High quality	H

**Table 6:** Sources quality evaluation results.

Quality Threshold	Accuracy	Completeness	Currentness	Quality Index
0, 0.5, 0.7, 0.9 and 1	0P 1L 43G 9H	29P 22L 2G 0H	1P 29L 20G 3H	0P 38L 15G 0H

**Table 7:** Quality and *shipments* incidents results according to the quality threshold.

Shipments Quality Threshold	Percentage of Quality Incidents	Percentage of Shipments Incidents
0	0	0.21
0.5	25	0.4
0.7, 0.9 and 1	21	0.3

**Table 8:** Shipments events number evolution.

Shipments Quality Threshold	Number of Shipments Without Any Event	Number of Shipments with at Least One Event
0	421	1631
0.5, 0.7, 0.9 and 1	821	1231

**Table 9:** Shipments quality evaluation results.

Quality Threshold	Accuracy (in %)	Completeness (in %)	Currentness (in %)	Quality Index (in %)
0	26P 1L 1G 72H	48P 29L 19G 4H	18P 30L 40G 13H	27P 16L 44G 13H
0.5, 0.7, 0.9 and 1	0P 0L 0G 100H	64P 19L 17G 1H	12P 32L 41G 15H	2P 47L 42G 10H

**Table 10:** Shipments consistency evaluation results.

Quality Threshold	Sensors Group	Consistency (in %)
0	{1, 2, 3}	0P 3L 21G 76H
	{11, 12, 13}	0P 0L 73G 27H
	{15, 16, 17}	0P 0L 74G 26H
	{49, 50, 51}	0P 0L 68G 32H
0.5, 0.7, 0.9 and 1	{1, 2, 3}	0P 0L 15G 85H
	{11, 12, 13}	0P 0L 62G 38H
	{15, 16, 17}	0P 0L 88G 12H
	{49, 50, 51}	0P 0L 81G 19H

## Data Availability

Publicly available datasets were analyzed in this study. This data can be found here: http://db.csail.mit.edu/labdata/labdata.html.
